# Editorial for Special Issue “Luminescent Colloidal Nanocrystals”

**DOI:** 10.3390/nano13030607

**Published:** 2023-02-02

**Authors:** Aleksandr P. Litvin

**Affiliations:** PhysNano Department, ITMO University, St. Petersburg 197101, Russia; litvin@itmo.ru

The field of luminescent colloidal nanocrystals and the numerous nanosystems based on them has recently made a rapid breakthrough from initial basic research to real applications and devices. Structures of this type have a number of unique properties that are important for applications. These properties can vary greatly for different classes of nanocrystals, but some of the most important of them can be generalized: the customizability of the desired physical and chemical characteristics, and convenient, functional processing. Further development in this industry still requires a fundamental understanding of the processes occurring in nanostructures, as well as the development of methods for their synthesis and modification, approaches to their use in various applications, and the design of devices based on these nanostructures. Research in this direction is extremely important for the development of many industries, including biomedicine, photonics, optoelectronics, sensing, quantum communications, “green” technologies, and others.

This motivated the formation of a Special Issue entitled “Luminescent Colloidal Nanocrystals”, which brought together seven research papers of broad specifics, from fundamental research into physical processes in quantum nanocrystals to the optimization of the architecture of real devices based on them.

More specifically, Babaev and co-workers [1] studied photoexcitation dynamics in thin films composed of reduced graphene oxide and PbS quantum dots, which are of great importance for near-infrared photodetection and solar harvesting. They proposed that, beyond a traditional charge transfer phenomenon, additional mechanisms govern the photoexcitation dynamics. Based on their study of the photoluminescent response, they theoretically and experimentally showed that Auger recombination and nonradiative energy transfer change the kinetics of photoexcitation in closed-packed films of reduced graphene oxide and PbS quantum dots. Skurlov and Yin et al. [2] investigated the influence of doping on linear and nonlinear optical properties of CsPbBr_3_ perovskite colloidal nanocrystals. They demonstrated that “B”-site doping ABX_3_ lead-halide perovskites induces drastic changes in fundamental optical characteristics, including photoluminescent quantum yield, radiative recombination rate, binding energy, and multiphoton absorption cross-sections.

The development of methods for the environmentally friendly, inexpensive, and convenient synthesis of new colloidal luminescent materials is the basis for their further application. To aid in this development, this Special Issue gathered research that details further progress in the synthesis of luminescent carbon-based nanomaterials. Carbon dots are a relatively new class of luminescent nanomaterials that has attracted significant research attention. Specifically, near-infrared emitting carbon dots may become a new technological platform for biomedical applications. Stepanidenko et al. [3] developed a template-assisted method for the synthesis of carbon dots emitting at 1085 nm. They showed that well-known organic dyes such as Rhodamine 6G and IR1061 may be used for the synthesis of carbon dots with new properties and tunable photoluminescent properties. Wu et al. [4] proposed a synthesis of chitosan-based green-emitting carbon dots. Importantly, they proposed a method of producing fluorescent composite films with high optical transparency in one pot that may be utilized for Fe^3+^ detection. Grudinkin et al. [5] studied another carbon-based luminescent nanomaterial, namely diamond nanocrystals, aiming for their further application in bio-sensing and labeling. They showed that post-synthetic treatment via reactive ion etching in oxygen plasma allows for the narrowing of their photoluminescence band due to the removal of surface sp^2^-induced defects.

The field of luminescent colloidal nanoparticles have demonstrated remarkably fast advancements, from basic research and synthesis to real applications. Interestingly, some colloidal nanoparticles may serve as multifunctional nanoobjects that make them promising for various areas including nanomedicine. Nigoghossian et al. [6] proposed the design of multifunctional iron oxide core–silica shell nanoparticles acting as both a magnetic heater and a self-referencing temperature emissive sensor. These multifunctional magneto-luminescent nanoparticles possess good thermal- and photostability and are promising for magnetothermia-related applications.

Optoelectronics is a particularly important area in the application of colloidal nanocrystals. The development of methods to synthesize, post-synthetically treat, and process luminescent colloidal nanoparticles made it possible to make a significant breakthrough in the creation of miniature emitters and detectors of optical radiation and photovoltaic devices. In line with this scope, Skurlov and Yin et al. [2] demonstrated that doping is a prospective strategy for improving the emissive characteristics of CsPbBr_3_ perovskite colloidal nanocrystals serving as an active layer of a light-emitting diode. Following this strategy, they built a green-emitting device with a peak external quantum efficiency of 10.6% and a peak luminance of 24,221 Cd·m^−2^. Moreover, colloidal quantum dots may serve as auxiliary layers in optoelectronic devices. Luo et al. [7] used MoS_2_ quantum dots to heal a bottom interface in a polycrystalline perovskite-based solar cell. They showed that MoS_2_ quantum dots allow for improvements in the perovskite crystallinity, passivate defects, and balance charge carrier transfer. As a result, a power conversion efficiency of as high as 19.95% was achieved.

In conclusion, we believe that the collected research papers will attract significant attention from a broad readership and will promote further extensive research in the field of luminescent colloidal nanocrystals.
